# Insurance Type and Menopausal Hormone Therapy Use Among US Women

**DOI:** 10.1001/jamanetworkopen.2026.23740

**Published:** 2026-07-17

**Authors:** Arina Chesnokova, Sunni L. Mumford, Allison Schachter, Rebecca F. Hamm, Scott Lorch, Matthew Klebanoff, Sarah Lindley, Erika Harness, Makeba Williams, Marilyn M. Schapira

**Affiliations:** 1 Department of Obstetrics and Gynecology, Perelman School of Medicine, University of Pennsylvania, Philadelphia; 2 Leonard Davis Institute of Health Economics, University of Pennsylvania Perelman School of Medicine, Philadelphia; 3 Department of Biostatistics, Epidemiology and Informatics, Perelman School of Medicine, University of Pennsylvania, Philadelphia; 4 Division of Neonatology, Department of Pediatrics and Center for Pediatric and Perinatal Health Disparities Research and Policy Lab, Children’s Hospital of Philadelphia, Philadelphia, Pennsylvania; 5 Department of Medicine, Perelman School of Medicine, University of Pennsylvania, Philadelphia; 6 Sidney Kimmel Medical College, Thomas Jefferson Department of Medicine, Perelman School of Medicine, University of Pennsylvania, Philadelphia; 7 Center of Innovation in Health System Research, Philadelphia VA Medical Center, Philadelphia, Pennsylvania

## Abstract

**Importance:**

Access to menopausal hormone therapy (MHT) in the US remains low. Understanding whether insurance type is associated with MHT underuse and racial disparities in menopausal care is critical for designing interventions that increase evidence-based and equitable menopause care.

**Objective:**

To examine whether Medicaid compared with private insurance is associated with lower MHT use among US women. A secondary objective explored the degree that payer type is associated with observed racial differences in MHT use.

**Design, Setting, and Participants:**

This cross-sectional study used the 2013-2014, 2015-2016, and 2017-2020 cycles of the National Health and Nutrition Examination Survey (NHANES) to identify a survey-weighted analytic subset of eligible respondents of a population-based survey of the US civilian, noninstitutionalized population. The study included women aged 45 to 64 years with Medicaid or private insurance coverage who were eligible for MHT. Statistical analyses were performed between March 26, 2024, and December 2, 2025.

**Exposure:**

Insurance type.

**Main Outcomes and Measures:**

MHT use was the primary outcome. A secondary analysis assessed whether insurance status was associated with observed racial differences in MHT use. Associations were estimated using multivariable logistic regression.

**Results:**

The unweighted sample included 1666 National Health and Nutrition Examination Survey respondents (n = 22 275 545, weighted). Of the weighted final sample, 91.4% had private insurance, and 8.6% had Medicaid. Individuals insured by Medicaid were less likely than those with private insurance to report a history of MHT use (10.7% [95% CI, 6.9%-16.0%] vs 20.7% [95% CI, 18.1%-23.5%]). After adjustment, Medicaid-insured women had lower odds of MHT use than privately insured women (OR, 0.50 [95% CI, 0.28-0.87]). In a secondary analysis, Black participants showed lower odds of MHT than White participants (OR, 0.68 [95% CI, 0.45-0.95]), but this difference was attenuated once insurance status was included in the model (adjusted OR, 0.72 [95% CI, 0.50-1.04]).

**Conclusions and Relevance:**

In this cross-sectional study of women at typical age for menopause, Medicaid coverage was associated with significantly lower MHT use, suggesting that insurance type may play a role in menopausal care disparities. Insurance status may also partially explain previously observed racial gaps in MHT use. These findings highlight the need for policy and clinical interventions to address insurance-related barriers and to promote equitable menopausal care.

## Key Points

**Question:**

Among US women in menopause without contraindications for menopausal hormone therapy, is insurance coverage type (Medicaid vs private) associated with use of menopausal hormone therapy (MHT)?

**Findings:**

In this cross-sectional study (1666 women; weighted, approximately 22 million), Medicaid coverage was associated with 50% lower MHT use compared with private insurance, even after adjustment for confounding variables. In secondary analyses examining race and ethnicity, insurance type, and MHT use, adjustment for insurance type attenuated the initially observed difference in MHT use between Black and White women.

**Meaning:**

This study found that insurance type was associated with MHT use and that differences in coverage may partially explain racial disparities in menopausal care.

## Teaser

This cross-sectional study examines whether Medicaid compared with private insurance is associated with lower menopausal hormone therapy use among US women.

## Introduction

Menopause is a physiological transition that typically occurs between 45 and 55 years of age in individuals born with functional ovaries who have not experienced conditions, medications, or surgical procedures affecting ovarian function.^1^ Although a natural part of aging for most women, the menopausal transition is often accompanied by a range of bothersome symptoms. These include vasomotor symptoms (VMS) such as hot flashes and night sweats, mood disturbances, sleep disruptions, changes in sexual function, and alterations in urinary continence, among others.^2,3,4,5^ In addition, ovarian aging has been associated with an increased risk of metabolic syndrome, cardiovascular disease, and cognitive decline.^6,7^ Among these symptoms, VMS are the most common, affecting up to 80% of individuals undergoing natural menopause.^8^

Menopausal hormone therapy (MHT) is considered the first-line treatment for bothersome VMS.^9^ However, following the release of the Women’s Health Initiative trial results in 2002 and 2004, rates of MHT initiation and prevalence decreased dramatically due to concerns regarding safety.^10^ Despite subsequent analyses of the Women’s Health Initiative trial and new data demonstrating that MHT is safe and effective for treating bothersome VMS in menopausal patients without contraindications and within 10 years of menopause onset, rates of MHT have remained low.^11,12^

Importantly, both the severity of VMS and the use of treatments vary by race and ethnicity.^13,14^ Individuals undergoing the menopausal transition who identify as Black or African American report more severe and frequent VMS, with an earlier onset and longer duration compared with other racial and ethnic groups.^15,16^ Despite experiencing more pronounced symptoms, Black patients tend to receive less treatment with MHT than their White counterparts.^15^ The underlying mechanisms for this treatment disparity are not fully understood.

One potential mechanism for low rates of MHT in menopause is insurance coverage. Insurance coverage in the United States significantly influences access to care, the quality of services received, and health outcomes.^17,18,19^ The contribution of insurance status to care delivery during menopause, particularly to the overall low use of MHT and disparities in treatment, remains unclear. Although US patients in menopause hold health coverage from a variety of sources, private insurance and Medicaid public insurance cover most insured women aged 45 to 64 years (76% have private insurance and 16% have Medicaid).^20^ Medicaid is hence the predominant public insurance for this age group. Notably, Medicaid covers a disproportionate number of minoritized people,^21^ which may affect the use of MHT among these populations.

This study aims to investigate whether there is an association between payer type—specifically Medicaid compared with private insurance—and the use of MHT among menopausal women who are candidates for therapy. In addition, the study seeks to investigate whether insurance type is assocated with the disparate treatment rates observed among menopausal individuals who identify as Black or African American.

## Methods

The institutional review board of the University of Pennsylvania waived protocol review because the data used in the study are publicly available and deidentified. The results are reported in accordance with the Strengthening the Reporting of Observational Studies in Epidemiology (STROBE) reporting guideline.^22^

### Data Source and Variables

We conducted a retrospective cross-sectional study using data from the National Health and Nutrition Examination Survey (NHANES) to assess the association between insurance type—specifically private insurance vs Medicaid—and the history of MHT use among women aged 45 to 64 years who were otherwise eligible for MHT.

For this analysis, we combined data from the 2013-2014, 2015-2016, and 2017-2020 NHANES cycles, such that the 2017-2020 cycle includes only prepandemic data. Detailed information on the NHANES design and sampling methodology is available elsewhere.^23^ In brief, NHANES is a continuous, repeated cross-sectional survey of the US civilian, noninstitutionalized population that uses a complex, multistage probability sampling design and survey weights. Data are collected through in-home interviews and standardized examinations in a mobile examination center. The NHANES protocol was approved by the National Center for Health Statistics Ethics Review Board, and adult participants provided written informed consent. Because each public-use cycle represents a cross-sectional sample, there are no repeated within-person measurements in this pooled analysis. While NHANES is designed to support nationally representative estimates for the US civilian, noninstitutionalized population, the present study reflects a restricted survey-weighted analytic subset defined by the study eligibility criteria.

Demographic and clinical data relevant to our research question were extracted from publicly available datasets provided by NHANES. The primary exposure was insurance status, categorized as either Medicaid or private insurance. A secondary exposure of interest was self-reported race and ethnicity, used in a secondary analysis to examine whether insurance status could explain the association between self-reported race and ethnicity and MHT use. In NHANES, race and Hispanic origin are collected in the home interview using the demographics questionnaire, and the released race and ethnicity variable is derived from responses to questions on race and Hispanic origin. For this study, Mexican American and other Hispanic were combined into a single Hispanic category. Participants were then categorized as Hispanic, non-Hispanic Asian, non-Hispanic Black, non-Hispanic White, and other non-Hispanic race, including non-Hispanic multiracial.

The primary outcome was the self-reported history of ever having used female hormones: “[Have you/Has sample person] ever used female hormones such as estrogen and progesterone? Please include any forms of female hormones, such as pills, cream, patch, and injectables, but do not include birth control methods or use for infertility.” We also examined additional variables related to menopause treatment collected by NHANES, including the use of supplements for menopause and having a prescription for menopause-related medications in the past 30 days.

### Inclusion and Exclusion Criteria

Participants were included if they self-reported “female” as their sex (“gender” was used in the question wording), were between 45 and 64 years of age, reported coverage by either Medicaid or private insurance, and were medically eligible for MHT based on their medical history responses (see exclusion criteria). We excluded participants who were pregnant at the time of the survey, those who had experienced natural or iatrogenic premature ovarian insufficiency, and those without insurance or covered by Medicare, a state insurance plan, or other government insurance. Participants with medical contraindications to MHT were also excluded (those with active liver disease; cardiovascular disease, specifically coronary heart disease, angina, myocardial infarction, and stroke; history of or current possible hormone-sensitive cancers including breast, ovarian, uterine, and brain). In addition, individuals missing data on the primary outcome were not included. All constructs on which inclusion or exclusion criteria and variables are based on and mapped to NHANES questions are included in eTable 1 in Supplement 1.

Certain key variables had a high rate of missing data despite imputation by NHANES. Notably, medical history variables that could preclude patients from MHT had a missingness of 85.1%. In these cases, if a respondent affirmatively indicated a condition making them ineligible for MHT, they were excluded from the analysis; however, respondents missing this information were included. Smoking status was another variable with a high missing rate (63.7% missing in the final cohort) and was incorporated into a combined cardiovascular disease variable. Other confounders with missing values after imputation, specifically nativity, hyperlipidemia, diabetes, reports of a healthy diet, and alcohol use, had a missingness of less than 2%, and complete case analysis was applied in the models.

### Statistical Analysis

Statistical analyses were performed between March 26, 2024, and December 2, 2025. All statistical analyses were performed using Stata, version 17 (StataCorp LLC).^24^ All statistical tests were 2-sided, with statistical significance defined as *P* < .05. Estimates are presented with 95% CIs where applicable. We pooled the NHANES cycles and applied annualized, weighted data to ensure representativeness of the US population.

Descriptive comparisons were conducted between respondents with private insurance and those with Medicaid, as well as for prior MHT users and nonusers. The primary analysis used a survey-weighted logistic regression to estimate the association between insurance type and prior MHT use, adjusted for race and ethnicity, educational achievement, nativity, cardiovascular risk factors (hypertension, hyperlipidemia, diabetes, and smoking), body mass index (calculated as weight in kilograms divided by height in meters squared), alcohol use, report of a healthy diet, and number of health care visits in the past year.

In the secondary analysis, to assess whether insurance type accounted for differences in MHT use by race and ethnicity, we fit 2 nested models in the full cohort: model 1 included race and ethnicity alone, and model 2 added insurance type. These were compared with the fully adjusted primary model, presented as model 3. We also conducted a formal adjusted mediation analysis specifying Medicaid coverage as the mediator, prior MHT use as the outcome, and non-Hispanic White participants as the reference group.

Race and ethnicity are socially constructed variables with no intrinsic biological basis, yet the interplay of individual and structural racism likely contributes to the documented disparity in the experience and treatment of menopause.^25^ Including race and ethnicity in regression models cannot fully disentangle the complex mechanisms of discrimination, cultural context, and historical inequities, yet it remains a practical approach to quantifying residual disparity after accounting for measurable confounders.^26^ Thus, we included self-reported race and ethnicity as variables in our analysis.

## Results

### Cohort

**Figure 1.**
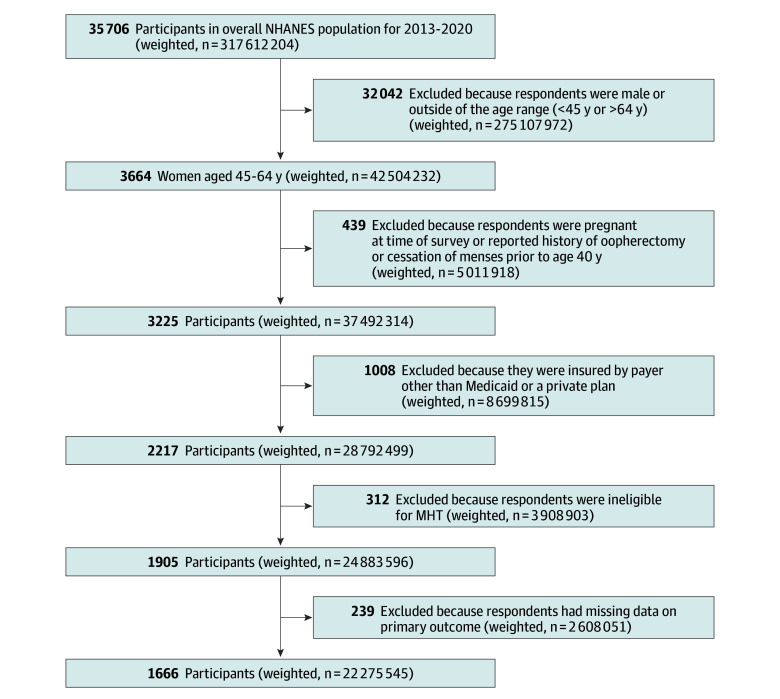
Flow Diagram Deriving the Cohort MHT indicates menopausal hormone therapy; NHANES, National Health and Nutrition Examination Survey.

The unweighted sample included 1666 NHANES responders (n = 22 275 545, weighted) (Figure 1). Prior MHT use decreased across cycles, from 25.1% in 2013 and 2014 to 16.7% in 2017 to 2020 (eTable 2 in Supplement 1). Of 1905 otherwise eligible participants, 239 (12.5%) had missing data on the primary outcome and were excluded from the complete-case analysis and were more likely to have Medicaid coverage, be born outside the United States, report limited English proficiency, and were less likely to identify as non-Hispanic White.

In our final weighted sample of respondents, 91.4% were covered by private insurance and 8.6% by Medicaid. In terms of race and ethnicity, 9.0% (95% CI, 7.0%-11.4%) covered by private insurance and 21.8% (95% CI, 16.5%-28.2%) by Medicaid identified as Hispanic, 5.1% (95% CI, 4.0%-6.5%) covered by private insurance and 7.3% (95% CI, 5.0%-10.6%) by Medicaid identified as non-Hispanic Asian, 9.2% (95% CI, 7.1%-11.8%) covered by private insurance and 25.5% (95% CI, 19.9%-31.9%) by Medicaid identified as non-Hispanic Black, 75.5% (95% CI, 71.3%-79.3%) covered by private insurance and 40.8% (95% CI, 32.7%-49.5%) by Medicaid identified as non-Hispanic White, and 1.2% (95% CI, 0.7%-2.2%) covered by private insurance and 4.7% (95% CI, 2.5%-8.5%) by Medicaid identified as belonging to other or multiple racial and ethnic groups (Table 1).

**Table 1.** Unadjusted Use of Medications and Supplements for Menopausal Symptoms, Along With Demographic and Clinical Characteristics Among Women Aged 45 to 64 Years Without Strict Contraindications to MHT, by Insurance Type and History of MHT Use^a^

Characteristic	Private insurance (n = 1391; weighted, n = 20 352 020)	Medicaid (n = 275; weighted, n = 1 923 526)	No past MHT use (n = 1378; weighted, n = 17 863 363)	MHT use in past (n = 288; weighted, n = 4 412 182)
Primary outcome				
History of MHT use for menopause, weighted % (95% CI)	20.7 (18.1-23.5)	10.7 (6.9-16.0)	NA	NA
Other prescription and supplement use indicators, % (95% CI)				
Used prescription medication for menopause in past 30 d	4.0 (2.6-6.2)	0.1 (0.0-0.5)	0.8 (0.3-2.0)	15.5 (10.3-22.7)
History of supplement use for menopause	5.2 (3.7-7.2)	1.0 (0.3-2.8)	4.2 (2.7-6.4)	7.5 (4.3-12.7)
Demographic characteristics				
Age, mean (95% CI), y	54.1 (53.7-54.6)	54.2 (53.3-55.2)	53.6 (53.0-54.1)	56.5 (55.8-57.2)
Race and ethnicity, % (95% CI)^b^				
Hispanic	9.0 (7.0-11.4)	21.8 (16.5-28.2)	10.6 (8.5-13.1)	8.1 (5.3-12.1)
Non-Hispanic Asian	5.1 (4.0-6.5)	7.3 (5.0-10.6)	6.0 (4.7-7.6)	2.5 (1.5-4.0)
Non-Hispanic Black	9.2 (7.1-11.8)	25.5 (19.9-31.9)	11.2 (8.9-14.0)	8.3 (5.9-11.4)
Non-Hispanic White	75.5 (71.3-79.3)	40.8 (32.7-49.5)	70.8 (66.3-74.9)	79.5 (73.9-84.1)
Other non-Hispanic race	1.2 (0.7-2.2)	4.7 (2.5-8.5)	1.5 (0.8-2.6)	1.8 (0.9-3.3)
Educational achievement, % (95% CI)				
Less than 8th grade	1.3 (1.0-1.9)	9.0 (6.0-13.3)	2.3 (1.7-3.2)	0.7 (0.3-1.6)
9-11th grade	4.0 (2.9-5.6)	21.9 (16.9-27.9)	5.8 (4.2-7.9)	4.9 (2.6-9.2)
High school diploma or GED	18.5 (15.8-21.6)	27.2 (21.4-33.9)	19.7 (17.0-22.8)	17.4 (11.9-24.8)
Some college	32.9 (29.1-36.9)	34.4 (27.0-42.8)	31.8 (28.0-36.0)	37.7 (31.2-44.7)
College degree or above	43.2 (38.7-47.9)	7.4 (4.7-11.4)	40.4 (35.4-45.5)	39.2 (33.1-45.7)
Nativity, % (95% CI)				
Born in the US	84.9 (82.3-87.2)	73.2 (64.6-80.4)	82.3 (79.4-84.8)	90.4 (86.7-93.1)
English proficiency, % (95% CI)				
Proficient in English	96.5 (95.5-97.3)	81.7 (76.4-86.0)	94.5 (93.2-95.5)	98.2 (96.9-99.0)
Clinical characteristics, % (95% CI)				
Presence of chronic conditions that should not impact MHT use^c^	11.8 (9.7-14.3)	14.7 (10.0-21.0)	11.2 (8.9-13.9)	15.6 (11.0-21.7)
CVD (presence of at least 1 CVD risk factor)^d^				
0 risk factors	40.0 (36.3-43.7)	20.7 (14.8-28.2)	40.0 (35.8-44.2)	31.5 (24.7-39.2)
1 risk factor	36.7 (33.2-40.4)	28.9 (23.5-34.9)	35.1 (31.3-39.2)	39.6 (31.5-48.3)
≥2 risk factors	23.4 (20.5-26.5)	50.4 (42.6-58.2)	24.9 (22.0-28.0)	28.9 (21.6-37.6)
BMI, % (95% CI)				
<30	63.6 (59.3-67.7)	49.5 (40.1-58.9)	61.9 (57.7-65.9)	64.3 (56.8-71.2)
≥30	28.3 (24.6-32.3)	39.4 (29.9-49.6)	29.6 (25.8-33.7)	27.9 (21.6-35.2)
≥40	8.1 (6.4-10.2)	11.1 (7.1-17.0)	8.5 (6.9-10.5)	7.7 (4.2-14.0)
Depression, % (95% CI)^e^				
Minimal to mild	94.7 (92.5-96.3)	72.4 (65.4-78.5)	93.0 (90.6-94.9)	91.7 (86.8-94.9)
Moderate	3.7 (2.3-5.9)	13.6 (8.9-20.3)	4.6 (3.0-7.0)	4.4 (2.4-7.9)
Moderately severe or severe	1.1 (0.6-1.9)	12.5 (8.6-17.9)	2.1 (1.5-3.1)	1.9 (0.9-4.0)
Lifestyle factors, % (95% CI)				
Reporting a healthy diet	78.8 (75.7-81.5)	49.7 (41.3-58.1)	73.9 (70.5-77.1)	85.6 (80.6-89.5)
Food insecure^e^	12.7 (10.5-15.2)	65.1 (58.4-71.2)	18.7 (16.1-21.6)	11.0 (8.1-14.9)
Heavy alcohol use^f^	43.2 (39.4-47.2)	33.1 (26.1-40.9)	42.4 (38.1-46.7)	42.4 (35.6-49.5)
Health care access and behavior, % (95% CI)				
Place of routine health care^e^	93.6 (90.8-95.6)	93.8 (88.3-96.8)	92.7 (89.4-95.0)	97.4 (93.8-98.9)
No. of visits in the past year^e^				
0	8.9 (6.6-11.9)	4.5 (2.4-8.2)	10.0 (7.3-13.5)	2.5 (1.0-6.1)
1-2	53.5 (49.3-57.6)	32.9 (26.6-39.8)	52.0 (47.9-56.2)	50.5 (42.7-58.3)
3-5	28.5 (25.6-31.6)	37.0 (28.8-46.0)	27.2 (24.3-30.3)	37.6 (30.3-45.5)
>5	9.1 (6.8-11.9)	25.7 (18.6-34.2)	10.8 (8.2-14.1)	9.3 (6.1-14.0)
Seen mental health care professional in past year, % (95% CI)	7.8 (5.9-10.2)	21.7 (15.0-30.3)	9.5 (7.2-12.5)	7.0 (4.9-9.9)

Abbreviations: BMI, body mass index (calculated as weight in kilograms divided by height in meters squared); CVD, cardiovascular disease; GED, General Educational Development; MHT, menopausal hormone therapy; NA, not applicable.

^a^ Pooled annualized 2013-2020 National Health and Nutrition Examination Survey (unweighted, N = 1666; weighted, N = 22 275 545).

^b^ Mexican American and other Hispanic were combined into a single Hispanic category. Participants were then categorized as Hispanic, non-Hispanic Asian, non-Hispanic Black, non-Hispanic White, and other non-Hispanic race, including non-Hispanic multiracial.

^c^ History of liver disease, chronic obstructive pulmonary disease, and non–hormone-sensitive cancer.

^d^ CVD risk factors include current hypertension, hyperlipidemia, diabetes, and smoking.

^e^ Variables with missing rates of 3% or less.

^f^ Heavy alcohol use was defined as 8 or more drinks per week on average.

Individuals insured by Medicaid were less likely than those with private insurance to report a history of MHT use (10.7% [95% CI, 6.9%-16.0%] vs 20.7% [95% CI, 18.1%-23.5%]), more likely to have lower educational attainment (with only 7.4% [95% CI, 4.7%-11.4%] holding a college degree or above compared with 43.2% [95% CI, 38.7%-47.9%] among privately insured), less likely to report a healthy diet (49.7% [95% CI, 41.3%-58.1%] vs 78.8% [95% CI, 75.7%-81.5%]), and more likely to experience food insecurity (65.1% [95% CI, 58.4%-71.2%] vs 12.7% [95% CI, 10.5%-15.2%]). Medicaid recipients were also more likely to identify as Hispanic (21.8% [95% CI, 16.5%-28.2%] vs 9.0% [95% CI, 7.0%-11.4%]) and non-Hispanic Black (25.5% [95% CI, 19.9%-31.0%] vs 9.2% [95% CI, 7.1%-11.8%]), have higher rates of obesity (body mass index ≥30: 39.4% [95% CI, 29.9%-49.6%] vs 28.3% [95% CI, 24.6%-32.3%]), and have 2 or more cardiovascular disease risk factors (50.4% [95% CI, 42.6%-58.2%] vs 23.4% [95% CI, 20.5%-26.5%]). In terms of health care use, a higher percentage of Medicaid-insured women had more than 5 health care visits in the past year (25.7% [95% CI, 18.6%-34.2%] vs 9.1% [95% CI, 6.8%-11.9%]). Those reporting a history of MHT use were older (mean age, 56.5 [95% CI, 55.8%-57.7%] years vs 53.6 [95% CI, 53.0%-54.1%] years), more likely to be non-Hispanic White (79.5% [95% CI, 73.9%-84.1%] vs 70.8% [95% CI, 66.3%-74.9%]), had higher educational levels, were more likely to be born in the US (90.4% [95% CI, 86.7%-93.1%] vs 82.3% [95% CI, 79.4%-84.8%]), and more likely to report a healthy diet and be less food insecure compared with those without past MHT use (Table 1).

### Use of Prescription Medications and Supplements for Menopause

**Figure 2.**
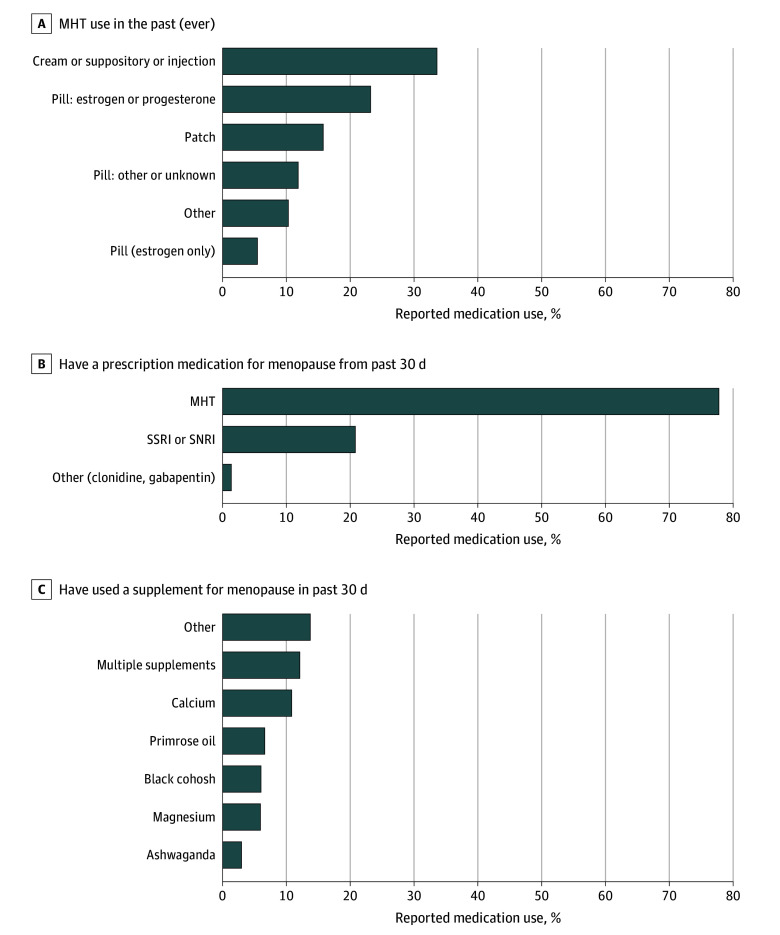
Bar Graphs Showing the Use of Medication and Supplements for Menopause Among Women Aged 45 to 64 Years Pooled National Health and Nutrition Examination Survey 2013 to 2020 cross-sectional estimate. A, Prescription medication use for menopause in the past 30 days; 3.7% of participants reported current prescription medication use for menopause, of which 77.8% was menopausal hormone therapy (MHT). B, Supplement use for menopause in the past 30 days; 4.9% of participants reported supplement use. C, Formulation among participants reporting prior MHT use; the most common formulations were pills (40.4%) and creams, suppositories, or injections (33.6%). Percentages are weighted estimates. SNRI indicates serotonin-norepinephrine reuptake inhibitor; SSRI, selective serotonin reuptake inhibitor.

**Figure 3.**
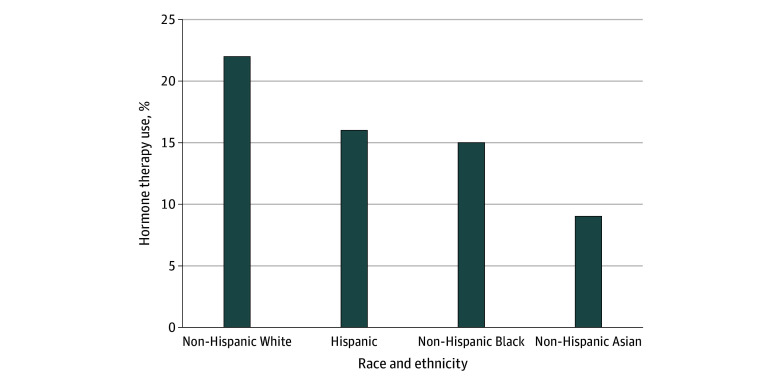
Bar Graph Showing Reporting of Hormone Therapy Use by Race and Ethnicity Pooled National Health and Nutrition Examination Survey 2013 to 2020 cross-sectional estimate.

In addition to self-reported history of MHT use, NHANES collected data on whether respondents had taken over-the-counter or prescribed medications for menopause in the past 30 days. Although 19.8% (95% CI, 17.2%-22.4%) of respondents reported prior MHT use, only 3.7% (95% CI, 2.1%-5.3%) had a current prescription for menopause; 77.8% (95% CI, 65.0%-90.6%) of these were for MHT. Among those reporting prior MHT use, the most commonly used formulation was pills (40.4% [95% CI, 33.4%-47.5%]), followed by creams, suppositories, or injections (33.6% [95% CI, 25.6%-41.5%]) (Figure 2). A higher proportion of respondents (4.9% [95% CI, 3.3%-6.4%]) reported using a supplement specifically for menopause in the past 30 days compared with 3.7% (95% CI, 2.1%-5.3%) who reported taking a prescription medication in the past 30 days. Weighted prior MHT use also varied by race and ethnicity, with the highest proportion among non-Hispanic White participants and lower proportions among Hispanic, non-Hispanic Black, and non-Hispanic Asian participants (Figure 3).

### Medicaid Coverage and Lower Odds of MHT Use

We assessed the association between insurance type and history of MHT use using logistic regression models adjusted for demographic and clinical characteristics. Women covered by Medicaid were significantly less likely to report a history of MHT use compared with those with private insurance (odds ratio [OR], 0.50 [95% CI, 0.28-0.87]) after adjustment for demographic and clinical characteristics (race and ethnicity, educational achievement, nativity, cardiovascular risk factors [hypertension, hyperlipidemia, diabetes, and smoking]), body mass index, alcohol use, report of a health diet, number of health care visits in the past year (model 3) (Table 2). Results were unchanged after additional adjustment for NHANES cycle (OR, 0.51 [95% CI, 0.29-0.89]) and in a missing-outcome sensitivity analysis assigning approximately 30.0% of missing outcomes to prior MHT use (OR, 0.51 [95% CI, 0.29-0.88]).

**Table 2.** Association Between Race and Ethnicity, Payer Status, and History of MHT Use^a^

	Odds ratio (95% CI)		
	Model 1: race and ethnicity as only covariate	Model 2: race and ethnicity and insurance status as covariates	Model 3: Race and ethnicity and insurance status as covariates, controlled for clinical and demographic factors^b^
Race and ethnicity^c^			
Non-Hispanic White	1 [Reference]	1 [Reference]	1 [Reference]
Hispanic	0.68 (0.45-1.01)	0.73 (0.49-1.09)	1.27 (0.78-2.04)
Non-Hispanic Asian	0.36 (0.22-0.60)^d^	0.38 (0.23-0.62)^d^	0.74 (0.36-1.50)
Non-Hispanic Black	0.68 (0.45-0.95)^d^	0.72 (0.50-1.04)	0.90 (0.56-1.43)
Other, including multiple races and ethnicities	1.08 (0.48-2.73)	1.23 (0.47-3.21)	1.52 (0.54-4.29)
Insurance coverage			
Private insurance	NA	1 [Reference]	1 [Reference]
Medicaid	NA	0.50 (0.32-0.78)^d^	0.50 (0.28-0.87)^d^

Abbreviations: MHT, menopausal hormone therapy; NA, not applicable.

^a^ Pooled annualized 2013-2020 National Health and Nutrition Examination Survey cross-sectional estimate (unweighted, N = 1666; weighted, N = 22 275 545).

^b^ Model 3 was adjusted for the following demographic and clinical characteristics: age, educational level, nativity, cardiovascular disease risk factors, body mass index, diet, alcohol use, and number of physician visits in the past year.

^c^ Mexican American and other Hispanic were combined into a single Hispanic category. Participants were then categorized as Hispanic, non-Hispanic Asian, non-Hispanic Black, non-Hispanic White, and other non-Hispanic race, including non-Hispanic multiracial.

^d^ Significant at *P* < .05.

### Race and Ethnicity, Insurance Type, and MHT Use

To investigate whether insurance type is associated with racial disparities in MHT use, we fit 2 nested models in the full analytic cohort and compared them with the fully adjusted primary model (Table 2). In model 1, which included race and ethnicity as the sole covariate, non-Hispanic Asian participants and non-Hispanic Black participants had lower odds of reporting MHT use compared with non-Hispanic White participants (OR, 0.36 [95% CI, 0.22-0.60] and 0.68 [95% CI, 0.45-0.95], respectively).

In model 2, after adding insurance status as a covariate, the difference between Black and White participants was attenuated (OR, 0.72 [95% CI, 0.50-1.04]), whereas Medicaid coverage was associated with lower MHT use (OR, 0.50 [95% CI, 0.32-0.78]). In the fully adjusted primary model (model 3), Medicaid coverage remained associated with lower MHT use, and no race or ethnicity category remained statistically significant. In a formal adjusted mediation analysis, for non-Hispanic Black participants, the indirect effect through Medicaid coverage was −0.009 (95% CI, −0.017 to −0.002; *P* = .01), whereas the direct effect after accounting for Medicaid was not statistically significant, consistent with insurance statistically accounting for part of the difference in ever MHT use between Black and White women (eTable 3 in Supplement 1).

## Discussion

In this nationally representative sample, 1 in 5 menopausal women (19.8%) (45 to 64 years old) reported ever using menopausal hormone therapy (MHT), yet only 3.7% had an active prescription for a medication to manage menopausal symptoms, most of which were MHT (77.8%).

Insurance status emerged as a significant factor associated with MHT use. Individuals covered by Medicaid had significantly lower odds (OR, 0.50 [95% CI, 0.28-0.87]) of having used MHT compared with those with private insurance. Furthermore, although initial unadjusted analyses showed an association between self-reported race and ethnicity and prior MHT use, this disparity was accounted for by insurance status in adjusted models, and this finding was confirmed in formal mediation analysis and suggests that insurance type may be a key factor associated with the observed racial and ethnic disparities in MHT use. Our study uniquely contributes to the literature by using pooled, nationally representative NHANES data, capturing actual US patterns to isolate the role of insurance as a structural determinant of systemic MHT use.

The rates of MHT observed in our study align with prevalence estimates from other large US-based cohorts, both overall and when stratified by race and ethnicity.^11,27,28^ For example, an analysis of the Study of Women’s Health Across the Nation (SWAN) cohort found overall initiation rates to be low (2.8%), with variations by race and ethnicity; approximately 4% among White women and less than 2% among Black women.^29^

Although Medicaid coverage has been associated in some studies with worse outcomes, such as higher readmission rates or complications, these findings often do not fully account for lower baseline health, socioeconomic disadvantage, and unmet health-related social needs among Medicaid enrollees.^17,18,30,31^ When these underlying differences are minimized, the picture is more nuanced. In narrow comparisons of adults with low income who are eligible for either Medicaid or marketplace plans (“on the cusp of eligibility”), Medicaid recipients have fewer office visits and more emergency visits yet do not consistently underperform on preventive care or medication adherence measures.^32,33^

The significant association between insurance status and MHT use in this adjusted analysis highlights the critical role of insurance in menopause care delivery, particularly for minoritized individuals. Investigating the mechanisms underlying this association is essential for addressing treatment gaps and disparities and improving health care outcomes for menopausal women.

Insurance appears to function as a mediator between race and ethnicity and treatment access, reflecting structural differences in coverage, reimbursement, and care delivery rather than individual-level preferences. Recognizing insurance as a modifiable, policy-relevant determinant opens tangible opportunities for intervention, such as expanding Medicaid formularies, improving reimbursement for menopause-related visits, and incentivizing equitable prescribing practices across payers.

Lower clinician participation rates in Medicaid may be another reason for these findings. Specifically, even when Medicaid-insured women in menopause do access care, the quality of the encounter may differ from that experienced by privately insured patients. The encounter may be shorter or the standard length of the encounter may not be adequate for a socially and medically more complex patient. Patients may experience less continuity with a health care professional, affecting trust building and acceptance of a particular treatment recommendation. The number of visits required to obtain a prescription for MHT may also pose a barrier, especially if scheduling appointments is challenging or if patients face logistical obstacles, such as transportation issues. Another reason for these findings may be clinician misunderstandings about coverage, such that clinicians may assume Medicaid excludes MHT or attaches high copays to its access.

A recent Oregon study by Rodriguez et al^34^ provides an important comparison for our findings. In a cross-sectional survey of 845 perimenopausal or postmenopausal individuals, the authors used the Menopause Rating Scale to assess symptom severity and found that 62.4% of those with moderate or severe symptoms were not receiving any therapy. After adjustment for age and rurality, public insurance was associated with higher odds of nontreatment (adjusted OR, 1.47 [95% CI, 0.99-2.19]). The most commonly reported reasons for nonuse were that therapy was not recommended by a clinician and concerns about safety or adverse effects. Although their outcome was treatment for menopausal symptoms broadly rather than MHT specifically, these findings are directionally consistent with ours and suggest that insurance-related differences in menopause care may reflect not only coverage itself but also differences in counseling, access to appropriately trained clinicians, and treatment decision-making.

Addressing the systemic issues underlying the Medicaid-associated disparity requires policy efforts that incentivize health care professionals to accept Medicaid and improve reimbursement rates to make serving Medicaid patients more financially viable. In addition, initiatives aimed at enhancing health care professional education on menopause, drug coverage, and cultural competence could improve the quality of care for Medicaid-insured women. Programs that facilitate care coordination and continuity, such as patient navigation services, may also help overcome barriers to accessing MHT.

### Strengths and Limitations

A major strength of this study is the use of NHANES data with appropriate weighting, which permits national estimates within the subgroup of US women who met the study eligibility criteria. In addition, NHANES collects detailed information on prescription medication use, including verification of current use, which improves the accuracy of our data regarding MHT use.

The study has several limitations. First, we were unable to assess the severity of VMS, a critical factor associated with both the need for and the use of MHT. The absence of data on VMS severity may bias our results toward the null, particularly because Black women who are menopausal may experience more severe VMS. In addition, there were limitations in the identification and coding of chronic conditions, which could affect the determination of medical eligibility for MHT. This study did not evaluate uninsured women, who may face distinct and potentially greater barriers to menopause care. We were also unable to assess rurality or other finer geographic barriers to menopause care due to the unavailability of geographic variables in the publicly available NHANES data.

Primary outcome missingness also warrants caution. Among otherwise eligible participants, 12.5% were missing the primary outcome, and those with missing outcome data differed from included participants on several observed sociodemographic characteristics, including insurance type, nativity, English proficiency, and race and ethnicity, suggesting that missingness was unlikely to be completely at random. In addition, missingness clustered across NHANES questionnaire components, suggesting partial module noncompletion. However, in a conservative sensitivity analysis in which approximately 30.0% of participants with missing outcome data were reassigned to ever MHT use, the association between Medicaid coverage and lower MHT use was materially unchanged. These findings reduce concern that complete-case exclusion alone explains the observed association, although selection bias from outcome missingness cannot be excluded. Also, the cross-sectional design and observational nature of the study limit our ability to draw causal links between insurance status and MHT use.

## Conclusions

In this cross-sectional study of MHT eligible women in menopause, our findings suggest that insurance status, specifically Medicaid coverage, is a significant factor associated with the low use of MHT. Although racial and ethnic disparities in MHT use are observed, insurance status appears to be a key factor associated with these differences. Addressing barriers associated with Medicaid coverage is essential for reducing treatment disparities and improving the health and quality of life for women experiencing menopausal symptoms. Further research is needed to explore targeted interventions that can enhance access to care and quality of care within this population.

## Notes

Supplement 1. **eTable 1. **Constructs from NHANES questions -> variables & inclusion and exclusion criteria **eTable 2.** Rates of reported menopausal hormone therapy (MHT) use, payer status, and demographic and clinical characteristics remained stable across the pooled NHANES cohorts **eTable 3**. Formal mediation analysis of the association between race and ethnicity and ever menopausal hormone therapy use, with Medicaid insurance coverage as mediator

Supplement 2.
Data Sharing Statement
