# Supplementary material for: Insurance Type and Menopausal Hormone Therapy Use Among US Women
Source: JAMA Netw Open. 2026 Jul 17;9(7):e2623740. doi: 10.1001/jamanetworkopen.2026.23740 (PMC13379748; doi:10.1001/jamanetworkopen.2026.23740)
Supplement: Supplement 1. — eTable 1. Constructs from NHANES questions -> variables & inclusion and exclusion criteria eTable 2. Rates of reported menopausal hormone therapy (MHT) use, payer status, and demographic and clinical characteristics remained stable across the pooled NHANES cohorts eTable 3. Formal mediation analysis of the association between race and ethnicity and ever menopausal hormone therapy use, with Medicaid insurance coverage as mediator [file jamanetwopen-e2623740-s001.pdf]

Supplemental Online Content

Chesnokova A, Mumford SL, Schachter A, et al. Insurance type and menopausal hormone therapy use among US women. *JAMA Netw Open*. 2026;9(1):e2623740. doi:10.1001/jamanetworkopen.2026.23740

eTable 1. Constructs from NHANES questions -> variables & inclusion and exclusion criteria

eTable 2. Rates of reported menopausal hormone therapy (MHT) use, payer status, and demographic and clinical characteristics remained stable across the pooled NHANES cohorts

eTable 3. Formal mediation analysis of the association between race and ethnicity and ever menopausal hormone therapy use, with Medicaid insurance coverage as mediator

This supplemental material has been provided by the authors to give readers additional information about their work.

© 2026 Chesnokova A et al. *JAMA Network Open.*

eTable 1. Constructs from NHANES questions -> variables & inclusion and exclusion criteria

Variable	NHANES variables by year	NHANES question	NHANES variable	Study variable construct
**Age**	2013-14: ridageyr<br>2015-16: ridageyr<br>2017-20: ridageyr	What is {your/{NAME}'s} birthdate?	Age in years of the participant at the time of screening.	Numerical age, no recoding
**Race and ethnicity**	2013-14: ridreth3<br>2015-16: ridreth3<br>2017-20: ridreth3	{Do you/Does SP} consider {yourself/himself/herself} to be Hispanic, Latino, or of Spanish origin? IF YES: Where {do your/do his/do her} ancestors come from? [Puerto Rican Cuban/Cuban American Dominican Republic Mexican/Mexican American Central/South American Other Latin American Other Hispanic or Latino]<br><br>What race do you consider {yourself/NAME} to be? Please select one or more. CHECK ALL THAT APPLY: AMERICAN INDIAN OR ALASKAN NATIVE ; ASIAN; BLACK OR AFRICAN AMERICAN; NATIVE HAWAIIAN OR PACIFIC ISLANDER; WHITE ; OTHER ; DK; RF	RIDRETH3 (Recode of reported race and Hispanic origin information, with Non-Hispanic Asian Category): 1 - Mexican American; 2 - Other Hispanic; 3 - Non-Hispanic White; 4 - Non-Hispanic Black; 6 - Non-Hispanic Asian; 7 - Other Race, including multi-racial	Combine 1 and 2 into "Hispanic" category to arrive at the following groups: Hispanic, Non Hispanic White, Non Hispanic Black, Non Hispanic Asian, Other, including multiple races and ethnicities
**Gender**	2013–14: RIAGENDR 2015–16: RIAGENDR 2017–20: RIAGENDR	"Was the sample person male or female?"	RIAGENDR (1 = Male, 2 = Female)	Restricted to "female" respondents; binary sex variable used as proxy for biologic sex.

© 2026 Chesnokova A et al. *JAMA Network Open.*

**Educational achievement**	2013–14: DMDEDUC2 2015–16: DMDEDUC2 2017–20: DMDEDUC2	"What is the highest grade or level of school {you have/SP has} completed or the highest degree received?"	DMDEDUC2: 1 = <9th grade, 2 = 9–11th, 3 = HS/GED, 4 = some college/AA, 5 = college grad or above	Categorical education variable; modeled as ordinal.
**Nativity**	2013–14: DMDBORN4 2015–16: DMDBORN4 2017–20: DMDBORN4	"In what country {were you/was SP} born?"	DMDBORN4: 1 = Born in 50 U.S. states/DC; 2 = Born in other countries/territories	Recoded as U.S.-born vs foreign-born.
**English proficiency**	2013–14: SIALANG / AIALANGZ 2015–16: SIALANG / AIALANGZ 2017–20: SIALANG / AIALANGZ	"Language used during interview/ACASI."	SIALANG (Interview language: English/Spanish); AIALANGZ (ACASI: English, Spanish, Asian languages)	Proxy for English proficiency (English vs other).
**Insurance coverage**	2013–14: HIQ011, HIQ031A–J, HIQ031AA 2015–16: HIQ011, HIQ031A–J, HIQ031AA 2017–20: HIQ011, HIQ032A–J, HIQ032AA	Lead question (HIQ011): "Are you covered by health insurance or some other kind of health care plan? [Include employer-based, direct purchase, Medicare, Medicaid, etc.]" Follow-up (HIQ031 series): "Are you covered by… (private insurance, Medicare, Medi-Gap, Medicaid, SCHIP, military, Indian Health Service, state-sponsored, other government, single service plan, etc.)"	HIQ011: 1 = Yes; 2 = No; 7 = Refused; 9 = Don't know. HIQ031A: Covered by private insurance (code = 14). HIQ031B: Covered by Medicare (15). HIQ031C: Medi-Gap (16). HIQ031D: Medicaid (17). HIQ031E: SCHIP (18). HIQ031F: Military/VA/Tricare/Champ-VA (19). HIQ031G: Indian Health Service (20). HIQ031H: State-sponsored health plan (21). HIQ031I: Other government insurance (22).	Study recode: Constructed as binary exposure: – *Private insurance* = HIQ031A. – *Medicaid* = HIQ031D. Excluded: Medicare (HIQ031B), Medi-Gap (C), SCHIP (E), military (F), IHS (G), state-sponsored (H), other government (I), single service (J), and uninsured (HIQ011=2 or HIQ031AA=40)

© 2026 Chesnokova A et al. *JAMA Network Open.*

			HIQ031J: Single service plan (23). HIQ031AA: “No coverage of any type” (40).	
**Premature ovarian insufficiency (POI) or iatrogenic menopause <45**	2013–14: RHQ031, RHD043, RHQ060, RHQ305, RHQ332<br>2015–16: same<br>2017–20: same	“Have you had at least one menstrual period in the past 12 months?” (RHQ031). If no: “What is the reason you have not had a period in the past 12 months?” (RHD043). “About how old were you when you had your last menstrual period?” (RHQ060). “Have you had both of your ovaries removed?” (RHQ305). “How old were you when your ovaries were removed?” (RHQ332).	RHQ031: 1 = Yes; 2 = No. RHD043: 7 = Menopause/change of life; 3 = Hysterectomy. RHQ060: Age at last period. RHQ305: 1 = Yes, both ovaries removed. RHQ332: Age at oophorectomy.	Excluded if last menstrual period or oophorectomy occurred <40 years old (premature ovarian insufficiency/iatrogenic menopause). Excluded those with hysterectomy if age <40. Remaining = eligible natural menopause population (45–64 years).
**HT contraindications (strict exclusions)**	2013–14: MCQ160 (heart disease, stroke), MCQ170l (current liver disease), MCQ220 (cancer) MCQ230 (cancer type)<br>2015–16: same<br>2017–20: same	“Has a doctor ever told you that you had congestive heart failure, coronary heart disease, angina, a heart attack, or a stroke?” (MCQ160B–F). “Do you still have a liver condition?” (MCQ170L). “Has a doctor ever told you that you had cancer or a malignancy of any kind?” (MCQ220), with cancer type follow-up (MCQ230) to identify breast, ovarian, uterine, brain cancers.	MCQ160B–F: 1 = Yes for CAD, MI, angina, stroke. MCQ170L: 1 = Yes, current liver condition. MCQ220 + MCQ230: 1 = Yes; flagged if type = hormone-sensitive cancer.	Excluded if history of: active liver disease; cardiovascular disease (coronary heart disease, angina, myocardial infarction, stroke); hormone-sensitive cancers (breast, ovarian, uterine, brain). These formed the strict exclusion criteria for MHT eligibility.
**Chronic conditions**	2013–14: MCQ160 (emphysema, chronic bronchitis, non hormone sensitive cancer)	“Has a doctor or other health professional ever told you that you had … [condition]?”	MCQ160 series: 1 = Yes; 2 = No.	Used as covariates in adjusted models. Conditions included: chronic bronchitis/emphysema, cancer (non–hormone-sensitive).

© 2026 Chesnokova A et al. *JAMA Network Open.*

	2015–16: same<br>2017–20: same			
**CVD risk factors (composite)**	2013–14: BPQ020, BPQ030, BPQ040a, BPQ050a (hypertension), BPQ080 (high cholesterol), DIQ010 (diabetes), SMQ040 (smoking) 2015–16: same 2017–20: same	Hypertension: "Have you ever been told by a doctor or other health professional that you had hypertension, also called high blood pressure?" (BPQ020); "Were you told on 2 or more different visits that you had hypertension?" (BPQ030); "Were you ever told to take prescribed medicine for high blood pressure?" (BPQ040A); "Are you now taking prescribed medicine for high blood pressure?" (BPQ050A).<br>Cholesterol: "Have you ever been told that your blood cholesterol level was high?" (BPQ080).<br>Diabetes: "Other than during pregnancy, have you ever been told you have diabetes or sugar diabetes?" (DIQ010).<br>Smoking: "Do you now smoke cigarettes?" (SMQ040).	BPQ020–BPQ050A: 1 = Yes to any (ever told, confirmed, advised medication, or currently taking).<br>BPQ080: 1 = Yes, high cholesterol.<br>DIQ010: 1 = Yes, diabetes.<br>SMQ040: 1 = Every day / Some days = current smoker.	Constructed as composite risk factor variable. CVD risk = 1 if ≥1 risk factor present (HTN, hyperlipidemia, diabetes, current smoker). Categorized as 0, 1, or ≥2.

© 2026 Chesnokova A et al. *JAMA Network Open.*

**BMI (self-reported)**	2013–14: WHD010 (self-reported weight, pounds), WHD020 (self-reported height, inches) 2015–16: same 2017–20: same	"How much do you weigh without clothes or shoes?" (WHD010). "How tall are you without shoes?" (WHD020).	WHD010/WHD020: Continuous, self-reported weight (lbs) and height (in).	BMI calculated as kg/m^2 from self-reported height and weight. Categorized as **<30**, **≥30**, and **≥40**. Used as categorical indicator of overweight/obesity severity.
**Depression (PHQ-9)**	2013–14: DPQ010–DPQ100 2015–16: same 2017–20: same	"Over the last 2 weeks, how often have you been bothered by the following problems?" (9 items covering anhedonia, low mood, sleep, fatigue, appetite, guilt, concentration, psychomotor change, suicidal thoughts).	DPQ010–DPQ090: 0 = Not at all; 1 = Several days; 2 = More than half the days; 3 = Nearly every day.<br>DPQ100: Functional impairment.	Summed to create PHQ-9 total score (0–27). Recoded into 5 categories:<br>1 = 0–4 → Minimal depression<br>2 = 5–9 → Mild depression<br>3 = 10–14 → Moderate depression<br>4 = 15–19 → Moderately severe depression<br>5 = 20–27 → Severe depression<br>Missing if PHQ-9 incomplete. Variable used for descriptive and adjusted analyses.
**Diet (healthy diet)**	2013–14: DBQ700 (diet quality self-report) 2015–16: same 2017–20: same	"In general, how healthy is your overall diet?"	DBQ700: 1 = Excellent, 2 = Very good, 3 = Good, 4 = Fair, 5 = Poor.	Collapsed to binary: healthy diet = Excellent/Very good/Good vs. not healthy = Fair/Poor. Reported as % endorsing healthy diet.

© 2026 Chesnokova A et al. *JAMA Network Open.*

**Food insecurity**	2013–14: FSDAD (household food security category) 2015–16: same 2017–20: same	U.S. Food Security Survey Module.	FSDAD: 1 = Full food security, 2 = Marginal, 3 = Low, 4 = Very low.	Binary recode: Food secure = Full (1); Food insecure = Marginal, Low, Very low (2–4).
**Alcohol use**	2013–14: ALQ120Q (frequency of drinking), ALQ120U (unit of time), ALQ130 (≥4/5 drinks per day) 2015–16: same 2017–20: same	"In the past 12 months, how often did you drink any type of alcoholic beverage?" (ALQ120Q/U). "In the past 12 months, on how many days did you have 4 or more drinks in a single day?" (ALQ130).	ALQ120Q/U: Reported frequency and unit. ALQ130: Days with ≥4 drinks/day (women).	Constructed binary indicator for heavy alcohol use: 1 = any report of ≥4 drinks on a day in the past 12 months (ALQ130 ≥1); 0 = no heavy drinking reported. Harmonized across survey cycles to account for differences in unit reporting.
**Place of routine healthcare**	2013–14: HUQ030 2015–16: same 2017–20: same	"Is there a place that you usually go when you are sick or need advice about your health?"	HUQ030: 1 = Yes; 2 = There is no place; 3 = There is more than one place.	Binary recode: Yes usual source of care (1, 2) vs No usual source (2).
**Prior healthcare use (12 months)**	2013–14: HUQ051 2015–16: same 2017–20: same	"During the past 12 months, how many times have you seen a doctor or other healthcare professional about your health at a doctor's office, clinic, hospital emergency room, at home or some other place?"	HUQ051: Reported count (0–99); 77 = Refused; 99 = Don't know.	Coded as categorical: 0, 1, 2-7, ≥8 visits. Used as indicator of prior healthcare use.
Medication and supplement use for menopause variables				

© 2026 Chesnokova A et al. *JAMA Network Open.*

**History of HRT use for menopause**	2013–14: RHQ540 2015–16: RHQ540 2017–20: RHQ540	“Have you ever used female hormones such as estrogen and progesterone? Please include any forms of female hormones, such as pills, cream, patch, and injectables, but do not include birth control methods or use for infertility.”	RHQ540: 1 = Yes; 2 = No; 7 = Refused; 9 = Don’t know.	Binary variable for ever use of menopausal hormone therapy (MHT). 1 = Yes (ever used female hormones for menopause); 0 = No. Primary outcome for the analysis.
**Supplement use for menopause**	2013–14: DSQIDS_H (product names), incl. dsdsupp (supplement name text) and dsd128aa (reason/flag you used to subset); 2015–16: same; 2017–20: same	“Have you taken any vitamins, minerals, herbal supplements, or other dietary supplements in the past 30 days?” "For what reason or reasons do you take {PRODUCT NAME}?} (For what reason or reasons did the doctor or other health professional tell you to take {PRODUCT}?)"	Verbatim documentation of supplement respondent stated being used for menopause	Descriptive, categorical variable built from product names
**Current use of prescriptions for menopause**	2013–14: RXQ_RX_H (Prescription Medications), linked to RXQ_DRUG 2015–16: RXQ_RX_I / RXQ_DRUG_I 2017–20: RXQ_RX_J / RXQ_DRUG_J	“Have you taken or used any prescription medicines in the past month?” (RXDUSE). For each medication: name recorded and coded to Multum Lexicon.	RXDDRUG / RXDDRGID: Text and Multum code for medication; up to 3 ICD-10-CM reason-for-use codes (RXDRSC1–3).	Flagged if participant reported any active systemic menopausal hormone therapy (estrogen alone or combined estrogen–progestin, excluding contraceptives). Derived from Multum drug class “Hormones/hormone modifiers” (therapeutic code 80) with manual review for menopause-specific indications. Binary: 1 = current MHT prescription; 0 = none.

© 2026 Chesnokova A et al. *JAMA Network Open.*

eTable 2. Rates of reported menopausal hormone therapy (MHT) use, payer status, and demographic and clinical characteristics remained stable across the pooled NHANES cohorts

Variable	2013-2014 cohort (respondent n=462; representative n=6,149,295)	2015-2016 cohort (respondent n=422; representative n=5,816,328)	2017-2020 cohort (respondent n=782; representative n=10,309,922)
Current use of MHT (past 30 days)	4.7	4.5	2.6
History of MHT use for menopause (%)	25.1	19.8	16.7
Medicaid insurance (%)	7.8	7.7	9.7
Demographic characteristics			
Age (mean, 95%CI)	54.2 (53.6–54.9)	53.7 (52.7–54.8)	54.3 (53.7–54.9)
Race and ethnicity (%)			
Hispanic	8.3	8.9	11.8
Non Hispanic White	77	72.6	69.7
Non Hispanic Black	9.5	12.6	10.1
Non Hispanic Asian	4.3	4.3	6.5
Other, including multiple races and ethnicities	1	1.5	1.8
Educational achievement (%)			
Less than 8th grade	2.5	1.9	1.8
9-11th grade	6.5	4.1	5.9
High school diploma/GED	18.8	15.9	21.5
Some college	32.9	32.7	33.2
College degree or above	39.3	45.4	37.7
Nativity (%)			

© 2026 Chesnokova A et al. *JAMA Network Open.*

Born in the US	87.9	87.7	79.3
English proficiency (%)			
Proficient in English	96.7	95.3	94.3
Clinical characteristics (%)			
Presence of chronic conditions that should not impact HRT use [a] (%)	7	10.1	16.2
CVD (presence of at least one CVD risk factor) [b]			
0 risk factors	34.1	39.4	40.1
1 risk factor	34.9	35.9	36.7
2+ risk factors	31	24.6	23.1
BMI			
<30	62.2	58.4	64.7
>=30	31.3	32	26.6
>=40	6.5	9.6	8.7
Depression (%)[c]			
Minimal to mild	90.6	95.1	92.8
Moderate	6	2.8	4.7
Moderately severe or severe	3.3	0.9	2
Lifestyle factors			
Reporting a healthy diet (%)	80.6	75.8	73.9
Food insecure (%)[c]	14.5	17.6	18.5
Heavy alcohol use (%)[d]	41.8	39.2	44.4
Healthcare access and behavior			
Place of routine healthcare (%)[c]	95.3	96.7	90.8
Number of visits in the past year (%)[c]			
0 visits	4.6	8.5	10.9
1-2 visits	52.1	45.8	54.8

© 2026 Chesnokova A et al. *JAMA Network Open.*

3-5 times	32.6	35.4	23.8
>5 times	10.7	10.3	10.5
Seen mental health provider in the last year	8.7	10.1	8.5

[a] History of liver disease, chronic obstructive pulmonary disease, non hormone sensitive cancer.

[b] CVD risk factors include: current hypertension, hyperlipidemia, diabetes, smoking.

[c] Variables with missing rates of 3% or less.

[d] Heavy alcohol use was defined as 8 or more drinks per week on average.

© 2026 Chesnokova A et al. *JAMA Network Open.*

eTable 3. **Formal mediation analysis of the association between race and ethnicity and ever menopausal hormone therapy use, with Medicaid insurance coverage as mediator**

Race and ethnicity category (vs non-Hispanic White)	Race/ethnicity → Medicaid coverage (a path)	Direct effect on MHT use (c′ path)	Indirect effect through Medicaid (a×b)	Total effect (c path)
Hispanic	0.065 (0.021 to 0.110); P=.004	0.036 (-0.030 to 0.102); P=.285	-0.005 (-0.010 to -0.0001); P=.047	0.031 (-0.034 to 0.096); P=.351
Non-Hispanic Asian	0.061 (0.011 to 0.111); P=.018	-0.026 (-0.100 to 0.049); P=.495	-0.005 (-0.010 to 0.001); P=.095	-0.031 (-0.104 to 0.043); P=.414
Non-Hispanic Black	0.124 (0.074 to 0.174); P<.001	-0.031 (-0.086 to 0.024); P=.274	-0.009 (-0.017 to -0.002); P=.012	-0.040 (-0.095 to 0.015); P=.154
Other non-Hispanic race, including multiracial	0.235 (0.095 to 0.374); P=.001	0.047 (-0.105 to 0.199); P=.541	-0.018 (-0.036 to -0.00001); P=.050	0.030 (-0.120 to 0.179); P=.697

Coefficients are from an adjusted survey-weighted generalized structural equation model with Gaussian family and identity link for both mediator and outcome; coefficients therefore represent absolute probability differences. The mediator was Medicaid coverage versus private insurance. Non-Hispanic White participants were the reference group for race and ethnicity. Adjusted model included age, education, country of birth, cardiovascular risk factors, body mass index category, heavy alcohol use, healthy diet, and healthcare utilization in the prior 12 months. The common Medicaid → MHT-use path was β=-0.076 (95% CI, -0.134 to -0.018; P=.011). The joint Wald test of all race/ethnicity-specific indirect effects was $\chi^2(4)$=6.69; P=.153.

© 2026 Chesnokova A et al. *JAMA Network Open.*
