# Supplementary material for: Insurance Type and Menopausal Hormone Therapy Use Among US Women
Source: JAMA Netw Open. 2026 Jul 17;9(7):e2623740. doi: 10.1001/jamanetworkopen.2026.23740 (PMC13379748; doi:10.1001/jamanetworkopen.2026.23740)
Supplement: Supplement 2. — Data Sharing Statement [file jamanetwopen-e2623740-s002.pdf]

Data Sharing Statement

Chesnokova. Insurance Type and Menopausal Hormone Therapy Use Among US Women.
JAMA Netw Open. Published July 17, 2026. doi:10.1001/jamanetworkopen.2026.23740

Data
Data available: Yes
Data types: Deidentified participant data
How to access data: Publicly available data was used for this study.
When available: With publication

Supporting Documents
Document types: Statistical/analytic code
How to access documents: arina.chesnokova@pennmedicine.upenn.edu - analytic code can be requested from first author.
When available: With publication

Additional Information
Who can access the data: The data is already publicly available.
Types of analyses: The data is already publicly available.
Mechanisms of data availability: The data is already publicly available.
